# Superior dislocation of the patella: a case report

**DOI:** 10.1186/1749-799X-4-29

**Published:** 2009-07-30

**Authors:** Xavier Cusco, Roberto Seijas, Oscar Ares, Jose R Cugat, Montserrat Garcia-Balletbo, Ramon Cugat

**Affiliations:** 1 Orthopedic and Trauma Surgery, Fundación García Cugat Hospital Quiron Barcelona – Spain

## Abstract

**Background:**

Superior dislocation of the patella is an uncommon condition that mainly occurs in knees with a high patella and medial femorotibial degenerative arthritis. There are no previous reports of this condition occurring in association with tibial valgus osteotomy. Case report: We report the case of a patient in whom vertical dislocation recurred twice at 4 months after tibial valgus osteotomy. To avert additional recurrence or new dislocations, the patient was treated surgically to remove the existing osteophytes. Conclusions: An arthroscopic approach was decided because of the lower associated morbidity and good results with this technique compared to open surgery.

## Introduction

Superior dislocation of the patella is rare; fewer than 20 cases have been reported in the English literature [1,2]. Patients with this condition often have a high patella and degenerative disease of the femoropatellar joint with osteophytes. There have been no reports of this condition in patients who have undergone valgus osteotomy of the tibia.

We report the first case of superior patellar dislocation related with prior tibial valgus osteotomy, which was resolved by orthopedic reduction and arthroscopic removal of the osteophytes that blocked the kneecap at the time of dislocation.

### Case

A 52-year-old woman with medial femorotibial degenerative arthritis of the knee underwent physiotherapy to strengthen the quadriceps musculature, without success. Because of persistent mechanical pain, femorotibial and femoropatellar arthroscopy was performed, with debridement of the medial meniscus, synovectomy, closed tibial osteotomy, and osthosynthesis with a plate. The patient progressed favorably, began to walk at 4 weeks, and was able to carry out daily activity at 6 weeks.

**Figure 1:**
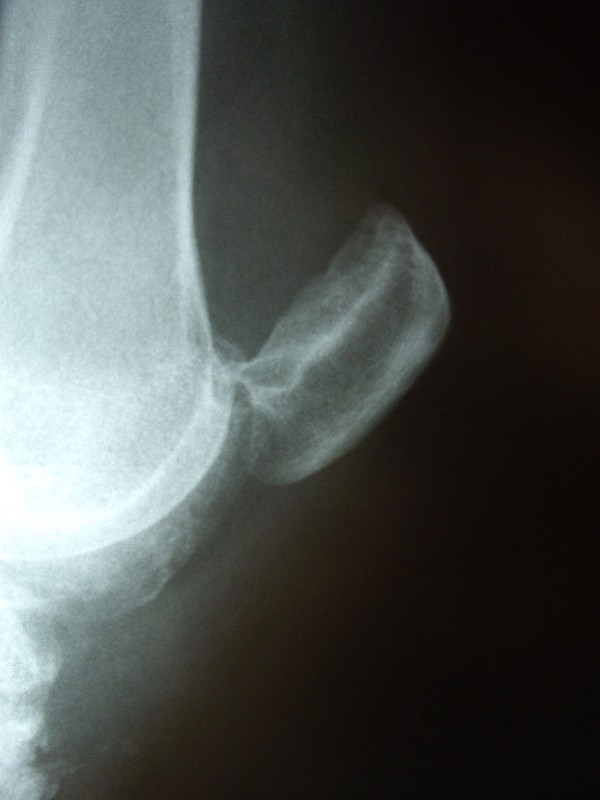
**Lateral radiograph showing the kneecap in superior dislocation with blocking in the osteophytosis of the inferior patellar pole and femoral osteophyte**.

**Figure 2:**
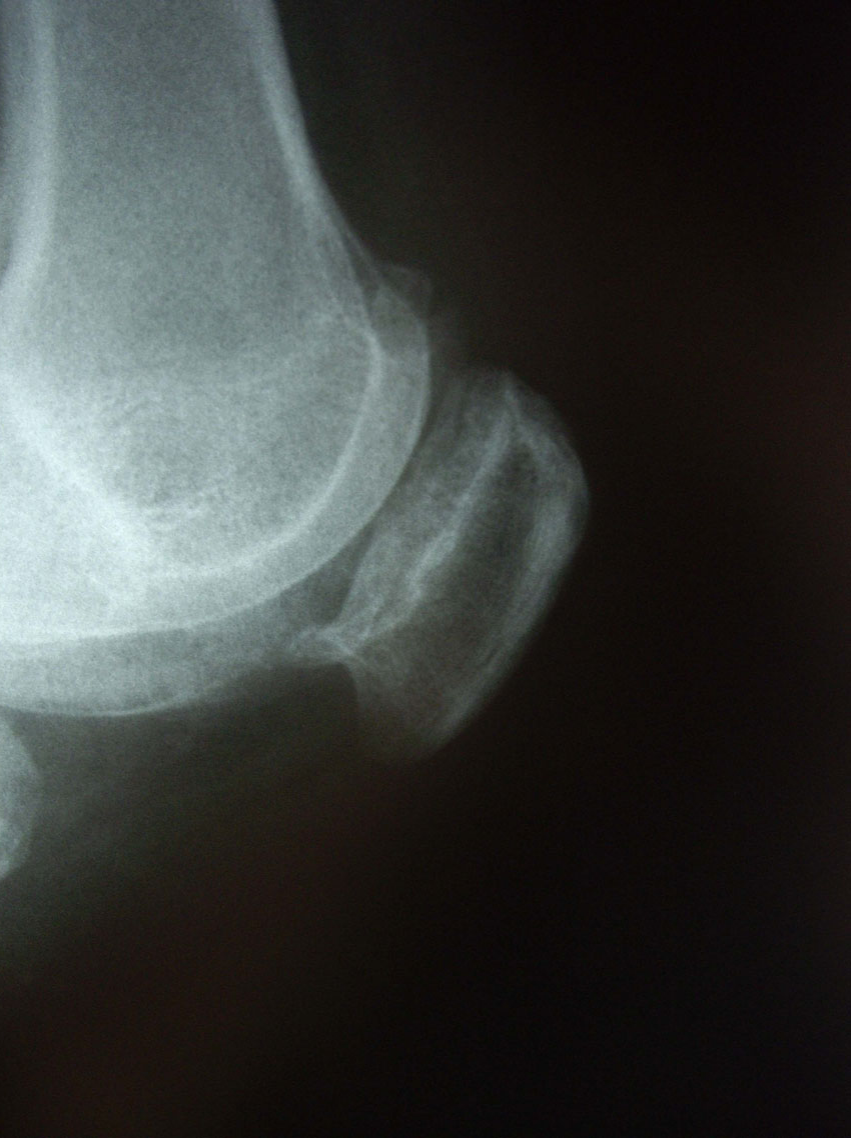
**Following orthopedic reduction, the presence of anterior femoral osteophytes is more evident**.

Four months later, she came to the emergency room after a sudden movement involving hyperextension, which resulted in pain and an inability to move the operated knee. Radiographs showed superior dislocation of the patella (figure 1), which was manipulated to obtain immediate orthopaedic reduction without the need for anesthesia. After reduction (figure 2), the patient experienced complete pain relief and restoration of joint mobility. Seventy-two hours later, she had another dislocation with self-reduction. Arthroscopic femoropatellar examination was then decided. The procedure, carried out 8 days later, revealed the presence of femoropatellar osteophytes in the area of the dislocation, which were completely removed. The patient progressed satisfactorily and had no new episodes of dislocation or knee problems over a follow-up of 2 years.

## Discussion

Superior dislocation of the patella has been reported in 18 patients, all except one with degenerative patellofemoral alterations [1,2]. Watson-Jones described the first case in 1956 [3,4]. The average age of presentation is 58 years (34–81), and logically, at a younger age it is less likely that patellar osteophytes are involved [5,6]. The single patient without degenerative disease was a 34-year-old man in whom the cause of superior patellar dislocation was a direct impact with hyperextension [2]. Our patient was 52 years old at presentation, which is consistent with the usual age range.

A high patella has been proposed as a predisposing factor [3,5,7] for this type of dislocation, as it is associated with several of the reported cases. The combination of a high patella and patellofemoral arthrosis constitutes a true risk factor because it favors the creation of free bodies [3,7] and osteophytes [5,8] at the inferior pole of the patella and the superior trochlea [9].

None of the reported cases have been related with previous tibial corrector osteotomy, as occurred in our patient, who underwent a valgus osteotomy of the tibia to treat degenerative disease of the medial femorotibial component. Occlusive osteotomy results in an increase in the patellar height.

The mechanism causing superior dislocation seems to be forced contraction of the quadriceps, with or without hyperextension of the knee [4,5,8,10]. Patellar dislocations are classified as intra-articular, which consists of rotation on the axial or vertical axis, or extra-articular, which can involve associated tendon rupture in addition to rotation [9]. In the reported cases of recurrent dislocation, the following risk factors have been described: high patella, ligament laxity, neurological alterations (paralysis), and previous severe genu recurvatum [4].

The differential diagnosis must be established with rupture of the patellar tendon [8,11]. In our patient there was an absence of patellar gap, palpable conservation of the patellar tendon [9], anterior leaning of the kneecap, and absence of a high patella, ruling out a ruptured tendon. Another entity that should be taken into account is vertical intra-articular dislocation.

Conventional radiography is the most common imaging study used in these patients. However, MR imaging is highly useful to visualize osteocartilaginous injuries [8] and is a helpful diagnostic test to rule out patellar ligament rupture [8].

Treatment for superior dislocation of the patella usually consists in local anesthesia and manipulation of the kneecap to unblock the osteophytes [1,2,8]. In recurrent cases, surgery has been proposed [6], involving arthroscopic debridement with removal of the osteophytes and chondral smoothing [3,5,8,10]. Some authors defend conservative management, even in recurrent cases [4]. Our patient had evident signs of degeneration at the site where the patella had blocked and two episodes of dislocation within 72 hours. We proposed arthroscopic surgery as a safe technique with less morbidity compared to open surgery to avert the risk of further dislocations. The osteophytes, which were in contact between the troclea and patella, were eliminated with an arthroscopic procedure.

## Consent section

Written informed consent was obtained from the patient for publication of this case report and accompanying images. A copy of the written consent is available for review by the Editor-in-Chief of this journal.

## Competing interests

The authors declare that they have no competing interests.

## Authors' contributions

Dr. X.C. and Dr. R.C. were the surgeons of the patient. Dr. JR.C and Dra. M.G-B were the revisors of the references. Dr. O.A and Dr. R.S conceived the paper and the writters of the paper. All the authors read and approved the final manuscript.
